# The Alcohol–High-Density Lipoprotein Athero-Protective Axis

**DOI:** 10.3390/biom10070987

**Published:** 2020-07-01

**Authors:** Corina Rosales, Baiba K. Gillard, Antonio M. Gotto, Henry J. Pownall

**Affiliations:** Houston Methodist Research Institute, 6670 Bertner Avenue, Houston, TX 77030, USA; crosales@houstonmethodist.org (C.R.); bgillard@houstonmethodist.org (B.K.G.); amg2004@med.cornell.edu (A.M.G.J.)

**Keywords:** alcohol, triglycerides, high-density lipoproteins, cholesterol, cardiovascular disease

## Abstract

Ingestion of alcohol is associated with numerous changes in human energy metabolism, especially that of plasma lipids and lipoproteins. Regular moderate alcohol consumption is associated with reduced atherosclerotic cardiovascular disease (ASCVD), an effect that has been attributed to the concurrent elevations of plasma high-density lipoprotein-cholesterol (HDL-C) concentrations. More recent evidence has accrued against the hypothesis that raising plasma HDL concentrations prevents ASCVD so that other metabolic processes associated with alcohol consumption have been considered. This review explored the roles of other metabolites induced by alcohol consumption—triglyceride-rich lipoproteins, non-esterified free fatty acids, and acetate, the terminal alcohol metabolite in athero-protection: Current evidence suggests that acetate has a key role in athero-protection but additional studies are needed.

## 1. Introduction

Plasma levels of low- and high-density lipoprotein-cholesterol (LDL-C, HDL-C) are positive and negative risk factors, respectively, for atherosclerotic cardiovascular disease (ASCVD). Although the former correlation has remained well supported, the salutary qualities of HDL, which have been widely cited, have now been called into question, as reviewed here, through studies in cells, animal models, and human interventional trials. Although statins and proprotein convertase subtilisin/kexin type 9 (PCSK9)-targeting drugs reduce plasma LDL-C concentrations, therapies that raise plasma HDL-C concentrations in a cardio-protective way are still needed to reduce residual ASCVD risk. Although past observational, prospective [1,2,3], and interventional studies [4,5,6] suggest that raising plasma HDL-C levels is athero-protective via multiple mechanisms, evidence against the raising-HDL-is-athero-protective hypothesis continues to accrue: (1) Not all patients with low HDL-C levels present with ASCVD and vice versa. (2) Patients with cholesteryl ester transfer protein (CETP) deficiencies have high plasma HDL-C levels but less cardio-protection than matched CETP-competent patients [7,8]. (3) Apolipoprotein A1 (APOA1) polymorphisms that elevate plasma APOA1 and HDL levels do not reduce ASCVD risk. [9] (4) Loss-of-function variants that raise (endothelial lipase) [10] or lower (adenosine triphosphate (ATP) binding cassette transporter subfamily A member 1 (ABCA1)) [9] HDL-C levels are not associated with reduced or increased ASCVD risk, respectively [9]. (5) Niacin therapy and CETP inhibition raise HDL-C but do not reduce ASCVD events [11,12,13,14]. Although niacin therapy reduced ASCVD in the Coronary Drug Project [15], in patients receiving a statin, niacin increased HDL-C from 35 mg/dL (0.91 mM) to 42 mg/dL (1.08 mM) but did not elicit a demonstrable ASCVD benefit in patients presenting with two components of metabolic syndrome (MetS)—hypertriglyceridemia and a low HDL-C concentration. [13] Thus, most HDL-increasing mechanisms are not athero-protective and, on a background of statin therapy, most HDL-raising interventions do not reduce ASCVD.

These observations provoked new hypotheses about possible mechanistic links between reduced ASCVD [16,17,18,19,20] and increased plasma concentrations of HDL-C, which has three proposed cardio-protective roles; HDL is a putative anti-oxidant, an anti-inflammatory agent, and an acceptor of macrophage free cholesterol (FC) efflux [21,22,23,24]. The anti-oxidant effects of HDL have been called into question because ~90% of the anti-oxidant capacity of plasma is afforded by other proteins that occur at much higher plasma concentrations—fibrinogen, immunoglobulin G, albumin, and the small, water-soluble molecules, uric acid and ascorbate, with albumin being the dominant anti-oxidant. HDL was a minor contributor (∼1–2%) to the anti-oxidant capacity of plasma. [25] The anti-inflammatory effects of HDL are likely indirect and are a cellular response to FC accretion [26,27,28]. Notably, macrophage cholesterol efflux (MCE) to HDL, reassembled HDL, or β-methylcyclodextrin reduces the number of inflammatory markers [29,30]. Thus, the HDL-mediated reduction in inflammation is an indirect consequence of MCE, the initial step in reverse cholesterol transport (RCT).

**Figure 1:**
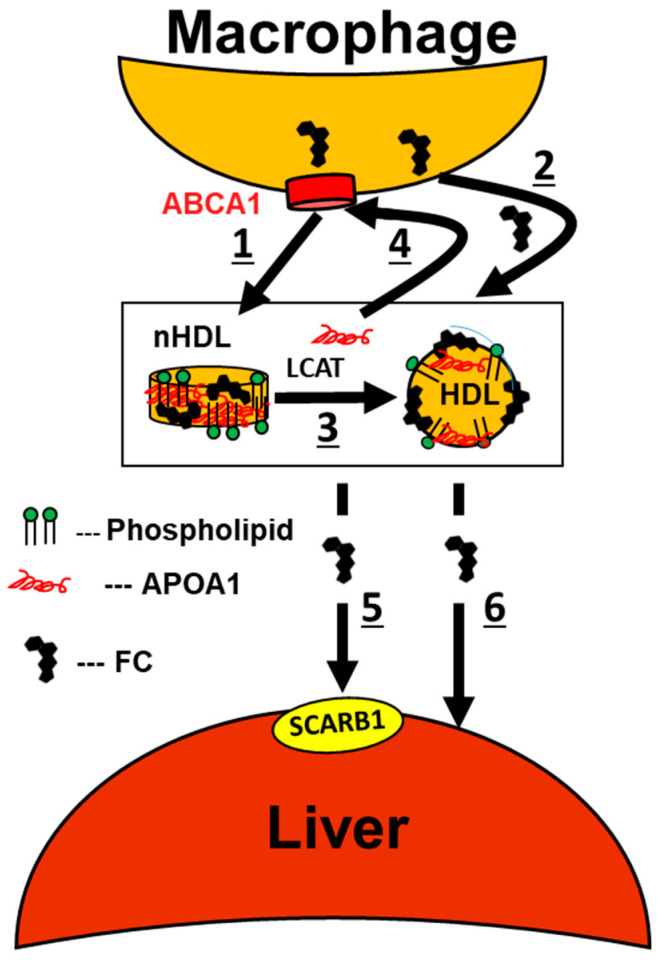
Traditional reverse cholesterol transport (RCT) model in the context of athero-protection. Nascent high-density lipoproteins (nHDL), apolipoprotein A1 (APOA1), adenosine triphosphate (ATP) binding cassette transporter subfamily A member 1 (ABCA1), free cholesterol (FC), lecithin:cholesterol acyltransferase (LCAT), scavenger receptor class B member 1 (SCARB1).

The current RCT model was formulated following the discovery of lecithin:cholesterol acyltransferase (LCAT), which converts FC to cholesteryl esters (CE). The authors reasoned that LCAT was required to reduce the cytotoxic effects of excess FC in peripheral tissue, including the arterial wall-macrophages where excess FC is likely atherogenic [31]. RCT comprises multiple steps (Figure 1): FC in arterial-wall macrophages transfers to early forms of HDL, i.e., nascent (n) HDL, via (1) the interaction of APOA1 with the ABCA1 transporter or (2) spontaneous FC transfer to nHDL and mature HDL. (3) LCAT esterifies nHDL-FC, which (4) promotes the extrusion of lipid-free APOA1 [32] for recycling to ABCA1. (5) FC and CE transfer to the liver via hepatic scavenger receptor class B member 1 (SCARB1). (6) FC also enters liver cells via spontaneous transfer. 

Formation of CE from FC via LCAT is important because this step converts FC, which transfers freely (minutes) among lipoprotein and membrane surfaces, to CE, which does not transfer spontaneously from HDL to peripheral tissue, and transfers slowly (hours) to other lipoproteins via CETP. Hypothetically, this prevents the reverse transfer of cholesterol into peripheral tissue including the arterial wall. Hepatic and steroidogenic tissue extraction of cholesterol via SCARB1 has a unique mechanism. Unlike the LDL receptor, which extracts the entire LDL particle, SCARB1 extracts only lipids [33,34] via a nibbling mechanism that leaves a protein-rich HDL remnant and lipid-free APOA1 [32]. Within the liver, FC is converted to bile acids that are excreted, thereby reducing the FC burden of peripheral tissue, including the arterial wall. In the absence of efficient RCT, cholesterol accumulates in arterial wall-macrophages, a process that precedes formation of cholesterol-rich atherosclerotic lesions. Thus, RCT, which is initiated by MCE, is integral to the athero-protective effects of HDL and APOA1. 

However, the extant data remain incomplete and are still debated because others report the opposite or no correlation between MCE and athero-protection [35,36,37,38,39,40]. Below, we reviewed the literature on the effects of alcohol ingestion on HDL metabolism and ASCVD with the goal of developing a refined mechanistic model linking HDL function to ASCVD. 

## 2. Correlating HDL, Alcohol Ingestion, and ASCVD

There are exceptions to the failed raising-HDL-is-athero-protective hypothesis. Both exercise and moderate alcohol consumption increase plasma HDL-C and -phospholipid (PL) concentrations [41,42,43,44,45,46,47], and reduce ASCVD risk [48,49,50,51,52,53]. Two drinks per day increase plasma HDL-C concentrations ~12% [54]. Whereas alcohol ingestion in the context of a typical Western diet induced a dose-dependent increase in HDL-C (≤18%), APOA1 (≤10%), and APOA2 concentrations (≤17%), the fractional clearance rates of APOA1 and APOA2 were not changed. These effects were associated with increased transport of APOA1 and APOA2 [55]. 

Whereas daily moderate alcohol consumption reduces ASCVD death in a dose-dependent way up to 1 drink/day, high alcohol intake increases death from other causes [56,57]. However, controlling for HDL-C does not affect the magnitude of the relationship between alcohol intake and ASCVD-related death (2011 Cohort of Norway) [58]. Alcohol intake does not affect expression of the major HDL-related genes coding for ABCA1, apolipoprotein A5 (APOA5), CETP, hepatic lipase, and lipoprotein lipase (CoLaus Study) [59]. Thus, the mechanistic links underlying the association between alcohol ingestion and reduced ASCVD remain to be identified—a complicated challenge given the broad effects of alcohol on multiple aspects of energy metabolism. Two hypotheses emerge about the cardio-protective effects of HDL—a direct effect on MCE and transport or a broader but more indirect effect on lipid metabolism. 

## 3. Alcohol Metabolism 

Alcohol has multiple pathophysiological effects on whole-body energy metabolism and the plasma lipoprotein profiles that originate in several hepatocyte-specific pathways [60]: Reduced gluconeogenesis lowers plasma glucose levels; lactate production induces plasma lactic acidemia; and sequential oxidation of alcohol forms acetaldehyde, a toxic compound that is then converted to acetate, which enters the plasma compartment. Alcohol-induced increases in hepatic triglyceride (TG) production have two pathological effects—some excess TG is secreted as very-low-density lipoproteins, thereby producing a hypertriglyceridemia, and unsecreted and non-degraded TG is hepatically retained and precedes fatty liver disease.

Given that the consumption of food and alcohol, usually together, are an embedded feature of many cultures, it is not surprising that both are also the subject of research in nearly every context of health and disease. According to 2016 World Health Organization statistics, per capita consumption of pure alcohol in the United States was ~9 L or about 25 quarts of 80-proof alcohol, a value that is slightly higher and lower than the average for men and women, respectively [61]. So, alcohol consumption makes a meaningful contribution to the diet of the average person and understanding its interactions with other environmental factors, especially food, and with genetic factors, is important to the rational design of public health strategies. Whereas alcohol consumption is associated with multiple aspects of lifestyle, meal, physical activities, smoking, culture, races, environment, and age, here we focused on the alcohol–HDL–ASCVD axis.

Hepatic alcohol metabolism is a short, linear, sequence of reactions—alcohol is converted successively to acetaldehyde, acetate, and acetyl-coenzyme A (acetyl-CoA) by alcohol dehydrogenase (ADH1B), acetaldehyde dehydrogenase (ALDH2), and acetyl-CoA synthase (ACSS2), respectively. Once formed, acetyl-CoA is a substrate for the citric acid cycle, ultimately producing cellular energy and releasing water and carbon dioxide. Genetic variants of these enzymes among individuals are associated with differences in catalytic efficiency and their capacity to metabolize alcohol. There are two major polymorphisms in the genes of alcohol metabolism that are relevant to the effects of alcohol on lipid metabolism and, perhaps, ASCVD. Among many East Asians, but not South Asians, acetaldehyde accumulates because they carry an allele of the gene coding for a super-activated alcohol dehydrogenase, a process that is exacerbated by the occurrence of a variant of the mitochondrial aldehyde dehydrogenase 2 variant 2 (ALDH2*2) allele. As a consequence, the pathway to acetate formation is impaired and plasma acetaldehyde concentrations increase, thereby inducing the “Asian flush” [62]. The impaired production of acetate is relevant to the function of many downstream effects of acetate, which enters multiple synthetic and regulatory pathways.

## 4. Chronic Alcohol Consumption Enhances MCE

With one exception [37], most large studies [21,22,63,64] found that MCE and HDL-particle number better predict reduced ASCVD than does HDL concentration. The role of particle number may be due to simple collision theory; during MCE, FC transfers to particle surfaces and a greater number of particles provides a greater number of surfaces. The correlation between MCE and reduced ASCVD suggests that the quality of HDL, i.e., its capacity to receive free cholesterol from cells, is different across patient populations and a more important ASCVD determinant than HDL concentration alone. This correlation provided the rationale for studies of the effects of alcohol consumption on MCE. Although the magnitude of the effect of MCE varied, all studies found that alcohol consumption by both male and female patients, in most studies ~40 g/day for more than two weeks as beer, spirits, or wine, increased plasma HDL-C and LCAT activity. More importantly, most studies showed that chronic alcohol consumption was associated with enhanced MCE from macrophages to patient plasma [65,66,67,68]. In most instances, the magnitude of MCE correlated with plasma HDL-C concentration. One study also found an attendant increase in HDL-phospholipid [69], likely an important MCE driver given that phospholipids are the essential FC-binding component of membranes and lipoproteins and the major compositional determinant of in vitro MCE [70,71,72], which occurs by both aqueous diffusion and via the macrophage FC transporter, ABCA1 [73,74].

## 5. Acute Effects of Alcohol Consumption

Although the acute effects of alcohol ingestion on MCE to plasma are not known with certainty, its effects on other aspects of lipid metabolism have been explored. Plasma chylomicrons, which form postprandially, are additional potential acceptors of cellular FC that is ultimately hepatically extracted [75]. Thus, it is expected that the lipemia produced by alcohol ingestion would also be associated with an increase in the MCE of plasma. However, the magnitude of alcohol-induced lipemia is much lower than that induced by an oral fat challenge and comprises mostly very-low-density lipoproteins (VLDL). The effects of consuming alcohol and fat together are expected to be different because preprandial alcohol synergizes with fat ingestion, profoundly increasing both the magnitude and duration of postprandial lipemia. Ingestion of alcohol (50 g) or fat (100 g corn oil) increases plasma triglyceride (TG) concentrations 50% and 100%, respectively. However, when consumed together, plasma TG concentration increases by 250%, a change that exceeds the sum of alcohol and fat consumed separately [76]. This observation was confirmed in another study showing that most of the increase in plasma TG concentration following the co-ingestion of alcohol and fat was due to chylomicron accretion with underlying inhibition of lipoprotein lipase [77]. MCE to whole postprandial plasma via the ABCA1- and SCARB1-dependent pathways is higher than those at baseline (+19 and 8%, respectively). The increase was associated with greater MCE to HDL_2_ than to HDL_3_ [78]. Other studies [79] have led to similar results, i.e., the postprandial increase in MCE is relatively small. The role of apolipoprotein B (APOB)-containing lipoproteins in MCE studies is usually ignored; most MCE studies are conducted with APOB-depleted plasma with the tacit assumption that HDL is the main determinant of MCE. However, another study revealed the importance of APOB-containing lipoproteins on MCE [80]. Although HDL is the initial (~20 min) acceptor of MCE, at later times the majority of MCE to plasma is associated with the APOB-containing lipoproteins, which, given their much faster turnover rates, would be expected to clear peripheral tissue-derived cholesterol faster than would HDL alone.

Acutely, alcohol ingestion also modifies the cholesteryl ester transfer activity (CETA) mediated by the cholesteryl ester transfer protein (CETP). Studies under conditions emulating normal-life alcohol consumption with an evening meal and under more strictly controlled clinical conditions gave similar results. In the first study, participants consumed food and alcohol as red wine during their evening meal. Wine consumption vs. mineral water increased postprandial plasma TG-rich lipoproteins’ concentrations and in vitro CE transfer from HDL to APOB-containing lipoproteins and transfer of TG in the opposite direction [81]. Plasma concentrations of total cholesterol, HDL-C and APOA1, APOA2, and APOB were unchanged. Thus, in a social setting, moderate wine consumption is associated with an enhanced exchange of VLDL-TG for HDL-CE.

Another study [82] correlated CETA with the plasma lipid concentrations among normotriglyceridemic (NTG) and mildly hypertriglyceridemic (HTG) volunteers who received alcohol, saturated fat (SAT), and alcohol + SAT in a controlled clinical setting. SAT (35 g/m^2^ body area) was consumed with and without alcohol (200 mL of a 10% weight for weight (*w/w*) solution) after an overnight fast. Over the following 10 h, bi-hourly samples were collected and analyzed. According to the area-under-the-curve (AUC) data, plasma cholesterol was unchanged over 10 h. However, alcohol induced an early decline in plasma non-esterified fatty acid (NEFA) concentrations due to inhibition of lipolysis of adipose tissue fat and plasma VLDL-TG, and recovered after ~4h. In contrast, SAT ingestion was followed by increased plasma NEFA due to lipolysis of the dietary fat. The NEFA response of alcohol + SAT was roughly the average of the two taken separately. Plasma TG modestly increased (5–10%) after alcohol ingestion but increased by 50 to 100% following ingestion of SAT and alcohol + SAT. Concurrently, plasma CETA increased as a function of plasma TG concentration following ingestion of SAT, a correlation that was more subdued by the combined alcohol + SAT ingestion. Thus, alcohol-mediated inhibition of HDL-CE transfer to chylomicrons maintains a higher plasma HDL-cholesterol concentration, which is possibly athero-protective. Although the suppressive metabolite underlying this correlation could be acetate, the terminal alcohol metabolite, other factors, including the CETA inhibitor, apolipoprotein F (APOF), are also likely important.

Thus, just as postprandial lipoproteins provide more acceptors for MCE, they also provide more acceptors for CETP-mediated transfer of CE from HDL, which are hepatically extracted faster than HDL. These findings are more generally applicable to persons who consume alcohol on a regular basis. Findings may be different when acute effects are compared among traditional abstainers and regular alcohol consumers because of the effects of alcohol on hepatic cytochrome P450 2E1 (CYP2E1) and cytochrome P450 3A4 (CYP3A4), which in humans work with the alcohol and aldehyde dehydrogenases [83].

## 6. The Role of Acetate in Alcohol-Mediated Effects on Plasma Lipid Metabolism

**Figure 2:**
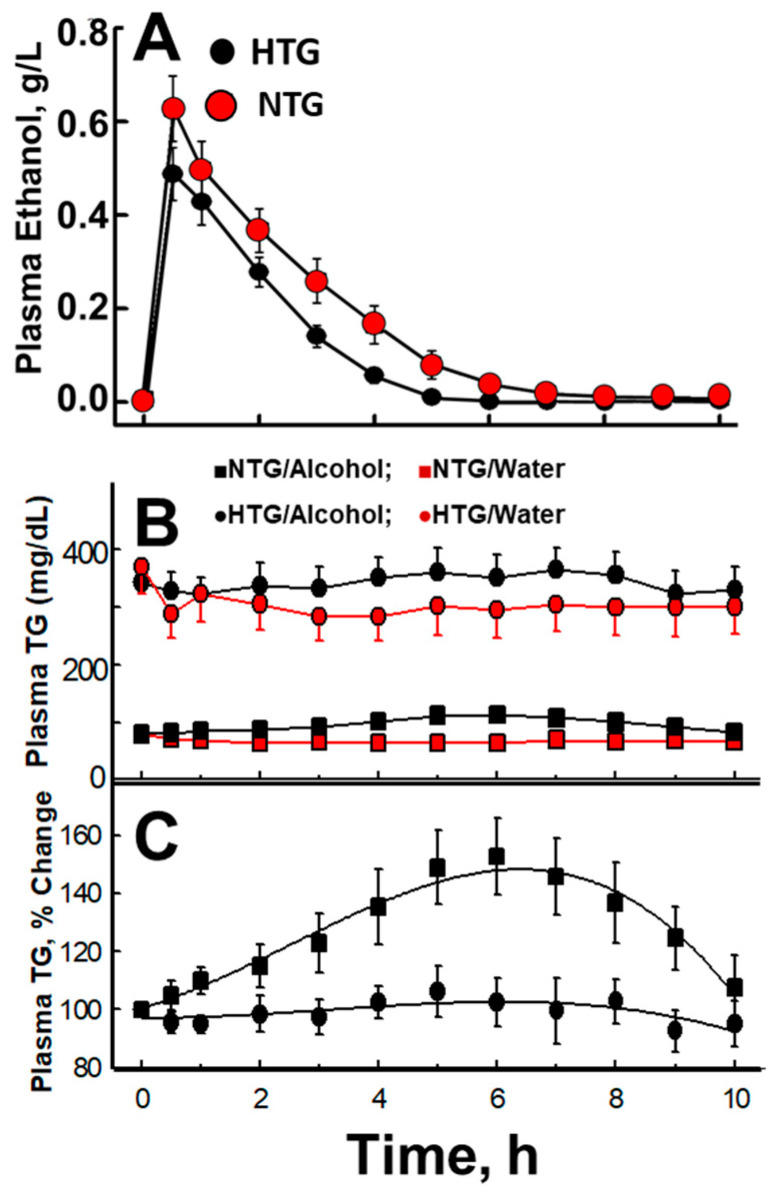
Plasma analytes among normotriglyceridemic (NTG) and hypertriglyceridemic (HTG) subjects after consuming 400 mL of 10% ethanol. (**A**) Ethanol concentrations, (**B**) Triglyceride (TG) concentration, and (C) % baseline triglyceride (TG); 6-h increases vs. baseline = 3% (HTG, *p* = 0.16) and 53% (NTG, *p* = 0.002). With permission [86].

**Figure 3:**
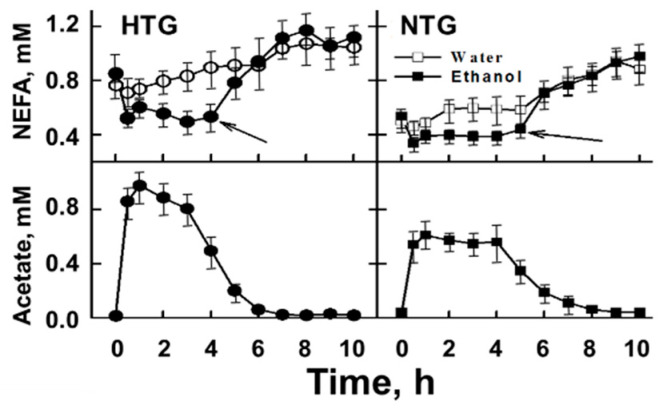
Plasma NEFA and acetate levels vs. time among HTG and NTG. Values are mean ± standard error of the mean (SEM). In both groups, the acetate concentration at which lipolysis recovers, according to increasing non-esterified fatty acid (NEFA) concentration, is ~0.2 mM acetate. With permission [86].

Whereas some researchers opine that alcohol, rather than any of its metabolites, is the essential determinant of its cardio-protective effects [84,85], clinical reports and recent studies in cells and genetically altered mice have implicated its terminal metabolite, acetate, in some key aspects of alcohol-determined cardio-protection. The physiological role of acetate in lipolysis was revealed by a study that compared the effects of alcohol (38 mL = 30 g of ethanol in 362 mL water in <15 min) on several relevant measures of alcohol metabolism among NTG and HTG volunteers [86]. The alcohol challenge produced the expected rise in plasma alcohol concentration to ~10 mM in both groups (Figure 2). Within the NTG group, there was a parallel 50% increase in the plasma TG concentration, whereas no significant change was observed among the HTG subjects. However, over the ensuing ~5 h, the plasma concentrations of NEFA, a marker of lipolysis, initially declined and then recovered (Figure 3).

Notably, the higher baseline NEFA levels among HTG vs. NTG volunteers is likely due to their higher rates of adipose-tissue lipolysis, which releases NEFA that are destined for hepatic TG synthesis and very-low-density lipoprotein secretion. Acetate metabolism is rapid; ingestion of 750 mg acetic acid/150 mL of water increases serum acetate (+150%) within 15 min, after which it declines with a half-time (t_1/2_) of ~15 min) [87]. The kinetics of alcohol-derived acetate are slower, rising to 0.9 and 0.5 mM, respectively, in HTG and NTG subjects, and then declining to 0.4 mM at ~5 h. At this concentration, increased plasma NEFA reveals the resumption of lipolysis. These observations implicate acetate (>0.4 mM) as an anti-lipolytic agent. However, the mechanism is not yet known.

Although no single study readily defines the role of alcohol-derived acetate in human lipid metabolism, there were early clues. Alcohol metabolism begins with its conversion to acetaldehyde and acetate via the successive activities of hepatic alcohol dehydrogenase and aldehyde dehydrogenase 2, respectively, the last reaction producing acetate, which is involved in many common metabolic pathways. Collective consideration of several studies suggests that acetate underlies the effects of alcohol on lipid metabolism. One early study compared the effects of alcohol, acetaldehyde, and acetate, alone or in combination, on lipolysis in isolated rat adipocytes in the basal and norepinephrine-stimulated states [88]. According to the rate of glycerol release, only acetate induced a dose-dependent inhibition of lipolysis. Inhibition of lipolysis by combinations of ethanol and acetaldehyde with acetate was similar to that of acetate alone. Thus, acetate alone was implicated in the inhibitory effects of alcohol on lipolysis observed in vivo.

The orphan G-protein coupled receptor, GPR43, was later identified as a second free fatty acid receptor (FFAR2) on leukocytes for which acetate and other short-chained fatty acids are ligands [89]. FFAR2 is also expressed in adipocytes, which, when treated with acetate, exhibit reduced lipolysis, an effect not observed in FFAR2^-/-^ vs. wild-type mice [90,91]. Moreover, FFAR2 activation by acetate reduces plasma free fatty acid levels, suggesting a potential role in broader aspects of normal lipoprotein metabolism and MetS. In humans, acetate and/or alcohol-derived acetate inhibit both adipose tissue (AT) lipolysis [88,92] and lipoprotein lipase (LPL) [77], leading to a profound lipemic response and dramatic, persistent decreases in the major lipolytic products, NEFA and glycerol. The anti-lipolytic effects of acetate in adipose tissue likely underlies the similar effects of alcohol [77,86], which is converted to acetate. In contrast, the mechanism underlying alcohol-mediated LPL inhibition is not known.

## 7. Mechanisms Underlying the Alcohol–HDL Athero-Protective Axis

Although the mechanisms linking alcohol consumption to reduced ASCVD are not immediately clear, there are several possibilities. One possibility is improved MCE to plasma. The acute lipemia produced by alcohol ingestion, especially when consumed with fat-containing food, provides more acceptors for MCE and for the rapid transfer of CE from a slowly metabolized FC pool in HDL (t_1/2_ ~3–5 d) to pools that are rapidly removed—VLDL and chylomicron and its remnants (t < 5 h). The importance of this mechanism seems low because a high plasma TG concentration correlates with a low HDL-C concentration [93], an ASCVD risk factor. On the other hand, regular, moderate (chronic) alcohol consumption increases plasma HDL, a longer-lived plasma pool of acceptors of MCE. Although compelling evidence is sparse and/or anecdotal, alcohol-derived acetate seems the stronger candidate as the agent that is the ultimate cardio-protective effector via improved insulin sensitivity. Acetate and alcohol reduce plasma glycerol and NEFA, an effect attributed to reduced lipolysis [92]. Given that acetate inhibits lipolysis in isolated rat adipocytes [88] via a FFAR2 mechanism [91] and that acetate has profound, cardio-protective effects that are independent of plasma HDL-C levels, plasma HDL-C concentrations are more likely a biomarker of cardio-protection than a mechanistic link. Several observations seem to reduce the importance of HDL in cardio-protection [80]: MCE to whole plasma better correlates with APOB and non-HDL-C concentrations than with HDL-C or APOA1; the magnitude of MCE to whole plasma of MetS patients is greater than that to normolipidemic patients; weight loss among MetS patients reduces the magnitude of MCE to their plasma to that of normolipidemic patients. Given the positive effects of weight loss on ASCVD events, weight loss-associated improvement in blood pressure, plasma TG, LDL-C, non-HDL-C concentrations, and insulin sensitivity must be more important than concurrent reduction in MCE. According to these observations, reducing the levels of the traditional ASCVD risk factors is more important than increasing MCE. In light of the studies reviewed here on the role of alcohol and acetate in vitro, in vivo, and in human clinical trials, elucidating the mechanisms by which acetate promotes athero-protection may provide additional/complementary therapies for ASCVD treatment.

## 8. A Mechanistic Model Links Alcohol Ingestion to a Cardio-Protective Plasma Lipoprotein Profile

**Figure 4:**
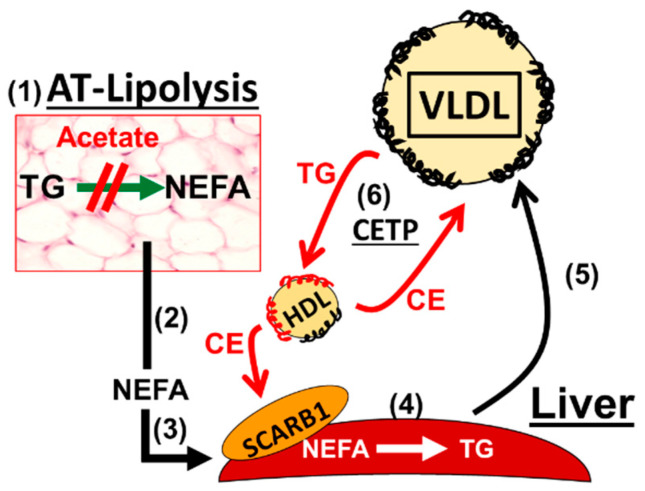
Mechanistic model for acetate-mediated alteration of the plasma lipoprotein profile (see text for details). Adipose tissue (AT), very-low-density lipoproteins (VLDL), cholesteryl ester transfer protein (CETP), cholesteryl ester (CE), non-esterified fatty acids (NEFA), high-density lipoproteins (HDL), triglycerides (TG), scavenger receptor class B member 1 (SCARB1).

The extant literature on alcohol and lipid metabolism [94] provoked our mechanistic model for the alcohol-mediated induction of a cardio-protective plasma lipoprotein profile (Figure 4). Adipose tissue lipolysis is an important determinant of the plasma lipoprotein profile. Under fasting conditions, the NEFA produced by adipose-tissue (AT) lipolysis (1) enter the plasma compartment (2) from which they are hepatically extracted (3). In the liver, the NEFA are esterified to TG (4), which are incorporated into VLDL that are secreted (5). In plasma, CETP exchanges the VLDL-TG for HDL-CE (6), thereby lowering the cholesterol content of each HDL particle and, with it, the plasma HDL-C concentration. Alcohol-derived acetate inhibits the first step, lipolysis, an effect that is followed by the inhibition of each successive step including the final step, reduction of HDL-C via the VLDL-TG for HDL-CE exchange, which leads to an elevation of plasma HDL-C. This model is supported in part by the negative correlation between plasma HDL-C and TG [93].

## 9. Acetate in Foods

Apple cider and wine vinegars, produced by fermentation of apple and grape juices, respectively, are in commonly consumed foods—salad dressings, marinades, vinaigrettes, and food preservatives. The sour taste of vinegar is due mostly to its content of acetate, which occurs as acetic acid. Whereas some opine that published clinical data claiming that apple cider vinegar can manage glucose, lipid levels, and weight loss are limited and equivocal, there is a large and convincing body of published studies to the contrary that cite several salutary properties of vinegar—reduction in the gastric emptying rate, improved insulin sensitivity, and reduced postprandial hyperglycemia among insulin-dependent diabetes mellitus patients [95,96,97,98]. Other studies, in normal and diabetic humans, revealed that acetate and/or vinegar ingestion is associated with a multitude of salutary effects, not all of which have been confirmed. These include weight loss, reduced plasma TG, total cholesterol, and LDL-C concentrations, but increased plasma HDL-C concentrations [99] and reduced blood glucose, insulin concentrations, and insulin resistance [100,101,102]. Vinegar also attenuated postprandial glycemia [98,103]. Human studies are supported in part by studies in rodents. In the context of high-fat diet (HFD)-induced metabolic disorders, vinegar ingestion by rats induces satiety and is hypolipidemic, hypoglycemic, anti-obesogenic [104,105], and anti-hypertensive [106], an effect that has not been tested in humans. In rats receiving a HFD, vinegar suppresses obesity-induced oxidative stress by modulating anti-oxidant defense systems and reverses HFD-induced obesity, hyperlipidemia, and oxidative stress [107]. Whereas studies in FFAR2^-/-^ mice show that FFAR2 directly mediates the stimulatory effect of acetate on insulin secretion and protects against islet apoptosis [108], the mechanistic connection between other salutary effects of vinegar/acetate remains to be determined. Thus, it appears that FFAR2 mediates many of the effects of acetate whether it is delivered as acetic acid or in numerous other forms including vinegar from multiple fruit sources. Moreover, acetate is also formed via colonic fermentation of acetogenic fiber [109,110] and inulin [111] and via the hydrolysis of triacetin, a common food additive.

## 10. Acetate as ASCVD Therapy

After alcohol ingestion, plasma acetate levels increase to millimolar concentrations [112,113]. Some speculate that ASCVD risk is lowered by the regular acetate intake in vinegar-and-oil salad dressings [106], so that the effects of alcohol on lipids, blood pressure, and ASCVD is mediated, in part, through its conversion to acetate and subsequent FFAR2 activation. Before the introduction of modern hypoglycemic agents, vinegar teas were used to control diabetes [106]. Small amounts of vinegar (~25 g) consumed with or without food reduce the glycemic index of carbohydrate-containing food for diabetic and non-diabetic persons [96,114,115]. This has been expressed as lower glycemic index ratings (~30%) [116,117].

**Figure 5:**
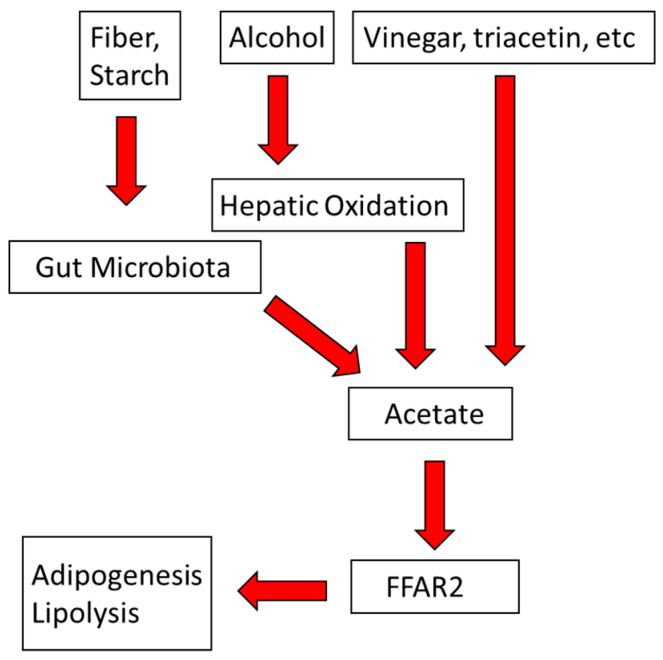
Schematic model of the conversion of dietary components into acetate, the bioactive ligand for free fatty acid receptor 2 (FFAR2).

Vinegar improves insulin sensitivity in normal and metabolic syndrome subjects [96,97], an effect that would be expected to reduce adipocyte lipolysis, thereby lowering plasma NEFA, hepatic TG production and secretion, and plasma TG levels [118]. Moderate daily alcohol intake is also associated with lower insulin secretion [56,57]. Thus, acetate from alcohol and other sources—dietary fiber, inulin, starch, which are converted to acetate by gut microbiota [112] — could be therapeutic via effects on AT-FFAR2 (Figure 5).

## 11. Open Questions

Alcohol consumption is associated with numerous pathologies—cancer, pancreatitis, fatty liver diseases, and obesity. The implication of acetate in alcohol-associated pathophysiology provokes several testable hypotheses as follows:

- Does acetate ingestion molar-equivalently enhance the postprandial lipemia seen with alcohol only?
- Whereas the lipemia induced by both alcohol and fat consumed separately increases with the magnitude of fasting plasma TG levels, is a similar relationship seen when acetate is consumed with fat-containing food and, if so, are the effects of acetate and fat co-ingestion synergistic or additive?
- Is the occurrence of obesity-linked diabetes, pancreatitis, and ASCVD among persons with mutation-associated deficiencies in alcohol-metabolizing enzymes different from that of those carrying the metabolically competent alleles?
- What if any role does the FFAR2 have in the etiology of ASCVD in consumers vs. non-consumers of alcohol and acetate or its precursors?
- Do molar-equivalent amounts of acetate and alcohol provide similar cardio-protective effects?

Answers to these question in humans and in the appropriate cell and animal models will better define the role of common food additives and possible acetate-based therapeutics in health and disease.
